# Stage-specific screening reveals differential resilience response to cold stress in rice

**DOI:** 10.1371/journal.pone.0338290

**Published:** 2026-04-15

**Authors:** Fahamida Akter, Partha S. Biswas, Abul Kalam Mohammad Aminul Islam, Mohammad Sharif Raihan, Md. Mizanur Rahman, Khandakar Md. Iftekharuddaula, Mohammad Rafiqul Islam, John Damien Platten

**Affiliations:** 1 Department of Genetics and Plant Breeding, Gazipur Agricultural University, Gazipur, Bangladesh; 2 Plant Breeding Division, Bangladesh Rice Research Institute, Gazipur, Bangladesh; 3 Department of Soil Science, Gazipur Agricultural University, Gazipur, Bangladesh; 4 International Rice Research Institute, Los Banos, Philippines; 5 IRRI Bangladesh Office, Dhaka, Bangladesh; Shahjalal University of Science and Technology, BANGLADESH

## Abstract

Rice cultivation in the northern and northeastern districts of Bangladesh faces cold stress during the seedling and reproductive stages that drastically reduces yield. As a precursor to generate improved elite lines, a diverse panel of rice germplasm was screened to identify genotypes with resilience to low temperature at both key developmental phases. Seedling-stage tolerance was assessed using an artificial cold-water tank, while reproductive-stage cold tolerance was evaluated under both natural field conditions and controlled cold screening facilities. Two breeding lines - BR8907-B-1–2-CS1–4-CS2-P3-4 and BR8909-B-12–2-CS1–4-CS2-P2-3–2, demonstrating minimal leaf discoloration and the highest survival rates, and lacking the known QTL (*qSCT1*) for seedling stage cold tolerance indicated contributions from other QTLs or genetic factors. Five genotypes (Bhutan, IR83222-F11-173, Rata Boro, BRRI dhan74, BR11712-4R-227) showed tolerance at the reproductive stage, while three lines (Bhutan, BR11712-4R-227, and BR12266-44-11-32-5-1-1-HR10-B) showed moderate tolerance at both seedling and reproductive stages, indicating stage-specific responses. The genotypes BR10317-5R-25, IR18A1859, and BRRI dhan28 were consistently susceptible at both stages. Principal Component Analysis (PCA) indicated that seedling and reproductive traits contributed jointly to variation under field conditions but independent under controlled environments, reflecting stage-specific genetic regulation. These findings highlight the complex, stage-dependent nature of cold tolerance and the importance of stage-specific screening to inform effective breeding strategies for enhanced cold resilience in rice.

## Introduction

Rice is the staple food for more than half of the world’s population, and is cultivated in over 100 countries. As the global population has already surpassed 7.5 billion and is projected to reach nearly 11 billion by 2100 [1], ensuring sufficient rice production has become increasingly important. In Bangladesh, rice contributes approximately 97% of total food grain production for a population of 172.92 million people [2]. By 2050, the population of Bangladesh is expected to reach around 215.4 million, requiring an estimated 44.6 million tons of milled rice to meet national demand [3]. Rice production, however, faces growing challenges due to declining arable land and increasing climate-related stresses, including drought, salinity, flooding, heat stress, cold stress and waterlogging. Among these constraints, low-temperature stress (LTS) is a major abiotic factor that limits growth, productivity, and yield of rice crop (S1 Fig in S1 File), affecting nearly 10% of the global rice-growing area (130 million ha) [4]. Rice cultivated during winter season in tropical and subtropical Asia is particularly sensitive to cold stress [5], especially during the seedling and reproductive stages [6,7]. In Bangladesh, approximately two million hectares of rice-growing areas are affected by low-temperature stress (LTS) each year [8].

In addition, the genetic makeup of rice has been optimized for warm environments through long-term domestication and selection process. Therefore, LTS is one of the primary abiotic stresses causing severe rice yield losses [9]. The optimum temperature range for normal growth and development of rice is between 25–30℃. Deviations beyond this critical range, rice plants are affected at variable degrees depending on the growth stage [10]. It can damage the plant during any developmental stage such as, germination, seedling, vegetative, reproductive and maturity [11]. Temperature below 20℃ significantly impair the germination process, reduce both the rate and percentage of germination, resulting in weak seedling establishment and poor crop stands [10]. During the winter season in northern Bangladesh, temperatures drop below 10°C at the seedling stage, causing leaf discoloration, yellowing, rolling, and stunted growth, often leading to seedling mortality and increased production costs [12]. Among all developmental phases, the reproductive stage is the most sensitive to cold stress and plays a major role in determining grain yield [13] Reproductive -stage cold stress thresholds are 20℃ and 15℃ for cold-sensitive and cold-tolerant varieties, respectively [14]. Reproductive development in rice comprises ten distinct stages, beginning with the initiation of panicle primordium and ending at flowering. Panicle initiation typically occurs approximately 30 days prior to flowering [10]. Cold stress during this period can have irreversible consequences. The most critical window of sensitivity occurs during the mid- reproductive phase, particularly at the young microspore stage, which takes place 10–12 days before heading. Exposure to low temperatures during this narrow developmental window induces male sterility [13], through malformed embryo sacs which inhibit pollen development and ultimately reduced spikelet fertility.

Cold stress during the booting stage disrupts pollen formation and leads to pollen abortion, resulting in no occurrence of anthesis. In contrast, when low temperature coincides with the flowering stage, pollen grain may form normally, but pollen tube germination is inhibited, leading to failure of fertilization. These cause panicle degeneration, incomplete panicle exsertion, and spikelet sterility, which ultimately lower the grain yield of rice [7,11,14–16]. These stage-specific disruptions ultimately cause high spikelet sterility and substantial yield losses [12]. Importantly, each reproductive sub-stage responds differently to cold stress, indicating that distinct physiological processes and regulatory mechanisms are involved [17].

The diverse and stage-dependent effects of cold stress reflect the involvement of complex and dynamic molecular pathways, including the differential expression of stress-responsive genes, hormonal signaling networks, and metabolic adjustments [9]. Because the physiological symptoms and underlying molecular mechanisms vary markedly from seedling to reproductive stages—and even among individual reproductive sub-stages, unique and stage-independent screening approach may fail to capture true genotypes having cold tolerance. Therefore, stage-specific cold stress screening is essential for accurately identifying tolerant genotypes capable of sustaining productivity under low-temperature stress conditions.

Screening of rice genotypes for cold tolerance under only natural field conditions may not be efficient because the natural climate is unpredictable in terms of its intensity, duration, and timing. The success rate of screening cold tolerance in natural field conditions is very low. This may be due to the complexity of cold stress tolerance traits, low genetic variance of yield components due to stress, and lack of proper selection criteria [18]. On the other hand, screening conducted under controlled (artificial) conditions may perform differently from actual field conditions. It’s, therefore, desirable to consider both field and artificial conditions when screening for tolerant genotypes [19]. This approach facilitates the large-scale identification and selection of cold-tolerant genotypes. Previous researchers used a reliable method of phenotyping for cold tolerance under both natural and artificial conditions [7]. The objective of this study was to screen for cold-tolerant genotypes by evaluating their performance at two key stages: the seedling stage, under controlled (artificial) conditions, and the reproductive stage, under both natural and artificial conditions.

## Materials and methods

### Plant materials

A diverse panel of 46 rice genotypes, including advanced breeding lines, local landraces, and BRRI varieties, was used in this study (Table 1). The materials were evaluated for cold tolerance at the seedling and reproductive stages. Seedling stage screening was performed using cold-water tanks and reproductive stage screening was performed in the phytotron facilities at Plant Breeding Division of the Bangladesh Rice Research Institute (BRRI), Gazipur. Reproductive stage cold screening was also performed under natural cold condition in the experimental fields of BRRI. A total of 34 genotypes and four check varieties were tested for seedling stage cold tolerance. Based on the seedling-stage screening results, a subset of genotypes was selected for further evaluation under cold stress at the reproductive stage. This subset included five genotypes showing susceptible reactions, four entries showing moderately susceptible reaction, and five genotypes showing moderately tolerant reactions. These selected genotypes were evaluated under cold stress at reproductive stage along with two known susceptible genotypes (IR18A1859, BRRI dhan28), two known tolerant genotypes (IR83222-F11-173, BRRI dhan67), four untested advanced lines, and four untested BRRI varieties.

**Table 1:** List of genotypes used in the study.

Gen._ID	Designation	Parentage	Types	Origin	Stages of cold screening
G1	Rata Boro		Local landraces	Bangladesh	SS and RS
G2	Tepi Boro		Local landraces	Bangladesh	SS and RS
G3	Mineasahi		Local landraces	Japan	SS and RS
G4	Bhutan		Local landraces	Bhutan	SS and RS
G5	IR83222-F11-173		Local landraces	IRRI	SS and RS
G6	Hbj. B. VI		Local landraces	Bangladesh	SS and RS
G7	IR100722-B-B-B-B-11		Advanced lines	IRRI	SS and RS
G8	IR100723-B-B-B-B-61		Advanced lines	IRRI	SS and RS
G9	BR10317-5R-25	BRRI dhan29/Hangangchal	Advanced lines	Bangladesh	SS and RS
G10	BR11303-5R-156	BRRI dhan28/HUA 565	Advanced lines	Bangladesh	SS and RS
G11	BR11318-5R-106	BRRI dhan29/HUA 565	Advanced lines	Bangladesh	SS and RS
G12	BR11712-4R-227	CN-6/BRRI dhan67	Advanced lines	Bangladesh	SS and RS
G13	BR12266-44-11-32-5-1-1-HR10-B	BRRI dhan28/N22	Advanced lines	Bangladesh	SS and RS
G14	BR8899-14-4-1-2-2-1	BR19/AS996	Advanced lines	Bangladesh	SS and RS
G15	TP16199		Advanced lines	Bangladesh	SS and RS
G16	IR18A1859		Advanced lines	Bangladesh	SS and RS
G17	BRRI dhan28	BR6 (IR28)/Purbachi	Variety	Bangladesh	SS and RS
G18	BRRI dhan67	IR61247-3B-8-2-1/BRRI dhan36	Variety	Bangladesh	SS and RS
G19	BR11894-R-R-R-R-169	BRRI dhan28/Bhutan	Advanced lines	Bangladesh	RS
G20	BR11894-R-R-R-R-329	BRRI dhan28/Bhutan	Advanced lines	Bangladesh	RS
G21	BR11894-R-R-R-R-105	BRRI dhan28/Bhutan	Advanced lines	Bangladesh	RS
G22	BR11894-R-R-R-R-110	BRRI dhan28/Bhutan	Advanced lines	Bangladesh	RS
G23	BRRI dhan74	BRRI dhan29/IR68144	Variety	Bangladesh	RS
G24	BRRI dhan88	BRRI dhan29-SC3-28-16-10-8-HR1 (com) (Somaclonal line of BRRI dhan29)	Variety	Bangladesh	RS
G25	BRRI dhan81	BRRI dhan28/Amol-3	Variety	Bangladesh	RS
G26	BRRI dhan92	Rice/Wheat (R1)/BR319-1-HR2//DH(Mingolo/Suweon290)/Panbira	Variety	Bangladesh	RS
G27	Rata Boro_Acc 8208		Local landraces	Bangladesh	SS
G28	Gochi Boro		Local landraces	Bangladesh	SS
G29	WANXIAN7777-P10		Local landraces	Bangladesh	SS
G30	Black rice		Local landraces	Bangladesh	SS
G31	BR69(Phyl)-P106		Advanced lines	Bangladesh	SS
G32	BR69(Phyl)-P107		Advanced lines	Bangladesh	SS
G33	7FBR-189		Advanced lines	Bangladesh	SS
G34	BR(Path)12452-BC3-42-22-11-4	BRRI dhan28/IRBL-9W	Advanced lines	Bangladesh	SS
G35	BR11315-5R-17	BRRI dhan58/HUA 565	Advanced lines	Bangladesh	SS
G36	BR8907-B-1–2-CS1–4-CS2-P3-4	BRRI dhan29/89010-TR1232-4-1-//BRRI dhan45	Advanced lines	Bangladesh	SS
G37	BR8909-B-12–2-CS1–4-CS2-P2-3–2	BRRI dhan28/CUNJING 15//BRRI dhan28	Advanced lines	Bangladesh	SS
G38	BR8938-30-2-4-2-1	IRBB60/BRRI dhan29	Advanced lines	Bangladesh	SS
G39	IR15L1691		Advanced lines	IRRI	SS
G40	IR19X1001:IRRI154-Cold1		Advanced lines	IRRI	SS
G41	IR19X1008:IRRI154-qSES1.2		Advanced lines	IRRI	SS
G42	IRRI154		Advanced lines	IRRI	SS
G43	BRRI dhan58	BRRI dhan29-SC3-28-16-4-HR2 (Somaclonal line of BRRI dhan29)	Variety	Bangladesh	SS
G44	BRRI dhan86	Niamat/BR802-78-2-1-1	Variety	Bangladesh	SS
G45	BRRI dhan89	BRRI dhan29*3/*Oryza rufipogon* (IRGC103404)	Variety	Bangladesh	SS
G46	BR1	IR262-24-3/TKM6	Variety	Bangladesh	SS

Sus. = Susceptible; Tol. = Tolerant; Mod. tol.: Moderately tolerant, SS: seedling stage, RS: Reproductive stage.

### Screening of cold tolerance at seedling stage

A total of 34 breeding lines were evaluated under artificial cold stress conditions at 13°C, using cold-water irrigation in a tank setup [20]. BRRI dhan67 and HBJ B VI were used as cold-tolerant check varieties, while BR1 and BRRI dhan28 were included as susceptible check varieties [21]. Each genotype, including the checks, was represented by 10 seedlings planted in single-row plots with 3.0 cm spacing. The seedlings were grown in flat-bottomed plastic trays (dimensions: 60 cm × 30 cm × 2.5 cm) containing fertilized soil free of crop residues and gravel. A total of 34 breeding lines, along with four check varieties, were evaluated using a row column design. The experimental units were arranged in trays, each of which was subdivided into two blocks, serving as blocks within the replication (S2 Fig in S1 File).

The experiment was conducted in three batches (Batch-1, seed sown on 6/22/2022; Batch-2, seed sown on 7/30/2022; Batch-3, seed sown on 7/24/2024) with four replications in Batch-1 and Batch-2 and six replications in Batch-3. A thin layer of finely granulated soil was applied after sowing to cover the germinated seeds. The seedlings were allowed to grow until the 3-leaf stage (8−12 days), (S3 Fig in S1 File) and then the trays were placed into the cold-water tank [22] that was pre-set at 13°C (S4 Fig in S1 File). In Batch-1, the temperature in the cold-water tank was not stable throughout the tank. The water temperature at six positions (four points at the four corners and two points across the middle of the tank) within the tank ranged from 10.3 to 14℃. Then, an aquarium internal filter motor (model: SOBO WP-1200F) of AC 220V 50 Hz 15 watts was installed into the tank to circulate the movement of water to stabilize the temperature close to 13℃ all over the tank. The temperature was recorded five times daily with an interval of 3 hours (6 AM, 9 AM, 12 PM, 3 PM, 6 PM) within the period of cold treatment (S5 Fig in S1 File). The duration of cold treatment across three batches ranged from 8 to 14 days. The water level was maintained at 2 cm above the soil level to keep the trays submerged. Cold stress symptoms were assessed using a subjective leaf discoloration (LD) scale ranging from 1 to 9, following the protocol outlined by Shim et al. [23] and IRRI [24] as shown in Table 2. LD scoring was conducted at the point when the susceptible check variety, BR1, exhibited complete mortality; normally this is done within six to ten days after cold treatment starts (Batch 1: 6 days, Batch 2: 14 days, Batch 3: 10 days) (S6 and S7A Figs in S1 File) [21]. After recording the LD score, the trays were placed at ambient temperature in a sunny place for recovery from cold stress. The survival count was recorded from each plot after a seven-day recovery period (S7B Fig in S1 File). The survival rate in percentage was calculated as the percentage of recovered green plants to the total number of plants used in the cold treatment.

**Table 2:** Leaf discoloration score for cold tolerance at seedling stage.

Scale for cold tolerance according to SES [23,24]	
1	No damage to leaves, normal leaf color (0–10% leaf tip dried)
3	Tip of leaves slightly dried, folded and light green, 11–30% leaf tip dried
5	Seedlings moderately folded and wilted, 31–50% seedlings dried, pale green to yellowish leaves
7	Seedlings severely rolled and dried; reddish-brown leaves (susceptible), 61–80% seedlings dried
9	Most seedlings dead and dying (highly susceptible), 81–100% seedlings dried

### Screening of cold tolerance at the reproductive stage

Screening of 22 diverse rice genotypes was carried out for reproductive stage cold tolerance along with three standard check varieties: BRRI dhan28 as susceptible, and BRRI dhan67 and IR83222-F11-173 as a moderately tolerant check and tolerant check, respectively. Screening was conducted under two different conditions: natural field conditions during the Boro season and artificial cold screening facilities (Phytotron) under cold stress and non-stress conditions.

### Field condition (natural condition)

To carry out field screening, one set of genotypes was sown on 21 October 2024 (34 days earlier than the regular sowing). This ensured the panicle initiation (PI) to booting stage of the crop would be exposed to low temperature from the end of January to the first week of February. This was the cold stress plot. A second set of the same genotypes was sown in the regular Boro season on 25 November and was treated as a non-stress plot. Flowering window and temperature profile of 25 genotypes were presented in S8 Fig and S9 Fig in S1 File respectively, for cold stress (A) and non-stress (B) conditions. Data on plant height, effective tiller number, days to heading, and panicle length were measured; vegetative stage score (VegS), panicle degeneration score (PDS), panicle exsertion score (PES), and spikelet fertility score (SFS) were recorded based on SES (Standard Evaluation System) presented in Table 3. The rate of reduction (RR) was calculated by the following method: RR = {(Mean value of nonstress – Mean value for cold stress)/mean value of non-stress} × 100%.

**Table 3:** Scoring details on cold injury-related traits [24].

Score	Data collected	Scoring scale
Vegetative stage	Maximum tillering stage	1 = Excellent, 3 = Good, 5 = Fair, 7 = Poor, 9 = Very poor: based on growth, tiller number and leaf color
Panicle degeneration	Flowering stage	1 = 0%; 3 = 1–10%; 5 = 11–25%; 7 = 26–40% 9 = > 40% of panicle degenerate
Panicle exsertion	Hard Dough stage	1 = > 1.0 cm, 3 = 0.5 - 1.0 cm, 5 = 0.0 - 0.5 cm, 7 = < 0.0 cm – 1/4th of the panicle, 9 = > 1/4th of the panicle length
Spikelet fertility	Hard Dough stage	1 = Highly fertile (>90%), 3 = Fertile (75–89%), 5 = Partly sterile (50–74%), 7 = Highly sterile (50% to trace), 9 = 0%

### Cold screening in phytotron

The same set of genotypes were evaluated in a artificial cold screening facilities (phytotron) with controlled air (15.9-19.14℃, average 17℃) and water (16.66-20.24℃, average 18℃) conditions, considered as cold stress (S10A Fig in S1 File) following a RCB design with 3 replications. The phytotron setup consisted of two water tanks, wherein a total of 100 entries can be accommodated for cold screening at the reproductive stage. Thirty-day-old seedlings of each genotype were transplanted

into six plastic pots with a single seedling per pot. Three pots were treated as cold stress, and three pots were used as a non-stress control. Plants were grown in ambient temperature in the net house until the meiotic phase of the reproductive stage began. The reproductive stage was determined by the distance between the ligule of the flag leaf and that of the penultimate leaf [10], considering an interval of −3 (flag leaf ligule below the penultimate leaf ligule) to +10 cm (flag leaf ligule above the penultimate leaf ligule) as indicative of this stage [21]. In the reproductive stage, three pots treated as cold stress pots were labeled and placed in the cold-water tanks in the phytotron for 10 days (S11 and S12 Figs in S1 File); the other three pots were kept at ambient temperature as a non-stress control. The temperature of air and water in the phytotron (S10A Fig in S1 File) and ambient temperature (S10B Fig in S1 File) was recorded five times in a day at 3-hour intervals. After 10 days of cold treatment, the cold stress pots were further placed at ambient temperature until the plants matured. A similar set of data, as well as field conditions, was collected from the labeled tillers for treated pots and six tillers from non-treated pots, and each panicle was individually harvested.

### Trait genotyping

Trait specific genotyping was performed with most cold-tolerant and most cold-susceptible rice genotypes identified from seedling and reproductive stage cold screening. Based on the screening results, a total of twelve genotypes at the seedling stage (two cold-tolerant, eight moderately tolerant, and two cold-susceptible) and eighteen genotypes at the reproductive stage (five tolerant, eight moderately tolerant, and five susceptible) were used for trait genotyping analysis. The response to cold stress of the most tolerant and susceptible genotypes at the seedling stage was validated using four SNP markers linked to the *qSCT1* locus, a previously reported QTL for seedling-stage cold tolerance (*snpOS00403*, *snpOS00404*, *snpOS00406*, and *snpOS00407*) derived from the Korean donor Gymbubyo (Table 6). The favourable alleles C:C, A:A, T:T, C:C for *snpOS00403*, *snpOS00404*, *snpOS00406*, and *snpOS00407*, respectively. Alternative genotypes at each locus represented as unfavorable allele. Similarly, the presence of reproductive stage cold tolerance-specific known QTLs *qPSST3* was monitored using SNP markers associated with *qPSST3* (*snpOS00815*, *snpOS00816*, *snpOS00817*), *qPSST6* (*snpOS01157*, *snpOS01158*), *qPSST7* (*snpOS00818*, *snpOS00819*, *snpOS00820*, *snpOS00821*), and *qPSST9* (*snpOS00822*, *snpOS00823*, *snpOS00824*, *snpOS00825*, *snpOS00826*). Favorable alleles were identified as G:G for *qPSST3_1*, *qPSST3_2*, and *qPSST3_3*; C:C for *qPSST7_1* and *qPSST7_4*; G:G for *qPSST7_2*; T:T for *qPSST7_3*; A:A for *qPSST9_1* and *qPSST6_1*; C:C for *qPSST9_2*, *qPSST9_3*, and *qPSST9_5*; and G:G for *qPSST9_4* and *qPSST6_2*. Alternative genotypes at each locus were considered unfavorable alleles.

**Table 6:** Genotyping results of qSCT1-associated alleles in cold-tolerant and cold-susceptible rice genotypes at the seedling stage.

ID	Designation	Status of seedling stage cold screening	qSCT1.1	qSCT1.2	qSCT1.3	qSCT1.4	Waxy
			snp OS00403	snp OS00404	snp OS00406	snp OS00407	snp OS00445
G36	BR8907-B-1–2-CS1–4-CS2-P3-4	Tolerant	T:T	C:C	G:G	T:T	C:C
G37	BR8909-B-12–2-CS1–4-CS2-P2-3–2	Tolerant	T:T	C:C	G:G	T:T	C:C
G4	Bhutan	MT	C:C	C:C	G:G	C:C	T:T
G6	Hbj. B. VI	MT	C:C	C:C	G:G	C:C	T:T
G11	BR11318-5R-106	MT	T:T	C:C	G:G	T:T	C:C
G12	BR11712-4R-227	MT	C:C	A:A	T:T	C:C	C:C
G13	BR12266-44-11-32-5-1-1-HR10-B	MT	T:T	C:C	G:G	T:T	C:C
G43	BRRI dhan58	MT	T:T	C:C	G:G	T:T	C:C
G45	BRRI dhan89	MT	T:T	C:C	G:G	T:T	C:C
G18	BRRI dhan67	MT	T:T	C:C	G:G	T:T	C:C
G17	BRRI dhan28	Susceptible	T:T	C:C	G:G	T:T	C:C
G46	BR1	Susceptible	C:C	A:A	T:T	C:C	C:C

MT: Moderately tolerant. Alleles highlighted in green and light pink represent favorable and unfavorable alleles for specific SNP, respectively.

### Data analysis

Descriptive statistics- including mean, range, standard deviation, and a two-stage analytical approach were employed in R version 3.2.1 to estimate LSD (least significant difference), heritability, BLUE (best linear unbiased estimation), and pBLUP (phenotypic best linear unbiased prediction) values. Grouping of genotypes by scatter plot was performed in Minitab.

Spearman’s rank correlation coefficient was analyzed using the cor.test () function. The formula for calculating the Spearman correlation is as follows:


$$\rho =\text{1}-\frac{\text{6}\sum \left(\overset{\text{2}}{\underset{i}{d}}\right)}{n\left(n^{\text{2}}-\text{1}\right)}$$ (1)

where ρ = Spearman correlation coefficient; di = the difference in the ranks given to the two variables’ values for each item of the data; n = total number of observations.

Principal component analysis (PCA) was performed using the prcomp() function. The grouping of germplasms was done by the hierarchical clustering method using Euclidean distance, and clustering was done by Ward’s minimum variance method (Ward.D2) via the hclust() function.

All analyses were performed using R statistical software (version 4.3.1) within the RStudio integrated development environment (IDE).

## Results

### Cold tolerance at the seedling stage

Leaf discoloration (LD) and survival percentage are the two important traits for discriminating cold-tolerant lines from susceptible lines under cold stress. The mean LD score recorded at 10 days of cold stress showed a wide range of variation among the genotypes (3.7 to 8.8), and the survival rate after seven days of recovery from cold stress also varied from 0 to 37% (Table 4).

**Table 4:** Summary statistics of Leaf discoloration (LD) and Survival Percentage in diverse panel of rice genotypes under seedling stage cold screening.

Parameter	LD	Survival %
Tested entries (n = 34)	3.7–8.8	0–37
BRRI dhan28 (Sus. check)	8.00	0.90
BR1 (Sus. check)	8.80	0.00
BRRI dhan67 (Mod. tol. check)	5.00	20.30
Hbj. B. VI (Tol. check_seedling)	5.70	17.40
p-value (Genotype effect)	3.02 × 10 ⁻ ⁸	2.04 × 10 ⁻ ⁴
CV (%)	12.59	29.97

The susceptible checks, BRRI dhan28 and BR1, exhibited high LD values (8.0 and 8.8, respectively) coupled with very low survival rates (0.9% and 0%). In contrast, the moderately tolerant check BRRI dhan67 and the tolerant check Hbj. B. VI recorded lower LD scores (5.0 and 5.7) and higher survival percentages (20.3% and 17.4%). Analysis of variance indicated a highly significant genotype effect for both traits (p = 3.02 × 10 ⁻ ⁸ for LD; p = 2.04 × 10 ⁻ ⁴ for Survival%). The coefficient of variation was 12.59% for LD, reflecting high experimental precision, whereas Survival% showed a CV of 29.97%, indicating substantial relative variability among genotypes.

**Fig 1:**
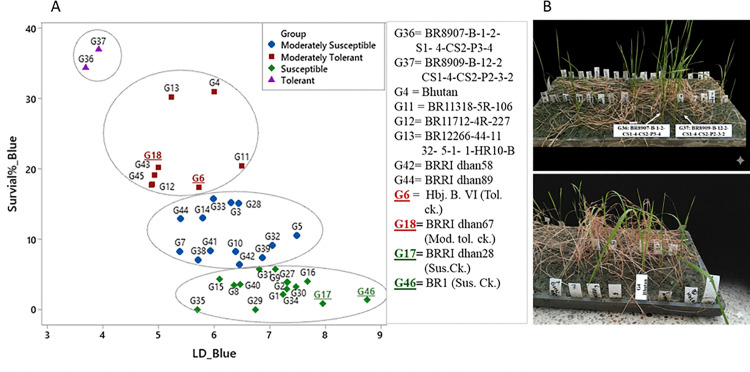
A) Clustering of genotypes based on BLUEs for LD (leaf discoloration) score and percent survival under cold water tank, B) Pictorial view of several seedling stage cold tolerant lines.

A scatter plot constructed based on combined BLUE for LD score and survival rate of 38 breeding lines, including check varieties, across the three batches presented in Fig 1A, and detailed data across three batches were presented in S1 Table in S1 File. Out of 38 genotypes, two genotypes, viz., BR8907-B-1–2-CS1–4-CS2-P3-4 and BR8909-B-12–2-CS1–4-CS2-P2-3–2, obtained the lowest LD score (3.7 and 3.9) and highest survival rate (34.4-37%) in all three batches, indicating these are highly tolerant at the seedling stage. Eight genotypes (Hbj. B. VI, Bhutan, BR11318-5R-106, BR11712-4R-227, BR12266-44-11-32-5-1-1-HR10-B, BRRI dhan58, BRRI dhan89, and BRRI dhan67) formed a moderately tolerant group having LD scores of 4.9-6.5 with 17.4-31% survivability. Pictorial view of several seedling stage cold tolerant lines was shown in Fig 1B. Thirteen genotypes were found as moderately susceptible (LD score 5.4-7.5 with survival 6.4-15.8%), and 15 genotypes showed susceptible (LD score 5.7-8.8 with survival 0-5.7%) to cold stress at seedling stage.

**Fig 2:**
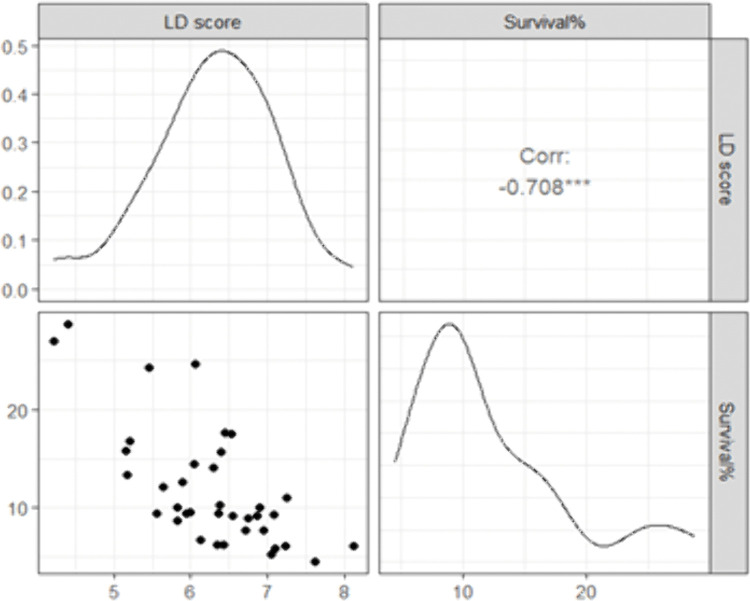
Distribution of cold tolerance phenotypes and Spearman correlation coefficients between LD score and survival% in a diverse panel of rice under cold stress in the cold-water tank.

The LD score exhibited the highest variability among the evaluated traits, with genotypes fitting an approximately normal distribution (Fig 2). Survival percentage, in contrast, showed a narrower distribution and was skewed towards lower values. A significant and negative correlation was observed between LD score and survival percentage (Spearman’s r = −0.708, p < 0.0001), indicating that genotypes with higher LD scores showed susceptibility to cold stress and tended to exhibit lower survival percentages.

An analysis of variance (ANOVA) was conducted to evaluate the effects of genotype, batch, and their interaction on leaf discoloration (LD) and survival percentage under cold screening conditions at the seedling stage (Table 5). A highly significant effect of genotype was observed both for LD and survival rate, indicating distinct LD and survival rate responses among genotypes. Batch and the genotype-by-batch interaction showed no significant interaction for LD and survival percentage, suggesting that the genotypes maintained their relative performance across different batches.

**Table 5:** Genotype-by-Batch interaction for the traits of leaf discoloration (LD) and percent survival under cold stress at the seedling stage in diverse rice genotypes.

Trait	Source	Df	Sum Sq	Mean Sq	F value	P value
LD	Genotype	37	447.20	12.09	5.94	<2e-16 ***
	Batch	1	8.50	8.47	4.16	0.06
	Genotype: Batch	35	81.90	2.34	1.15	0.26
	Residuals	380	773.70	2.04		
Survival%	Genotype	37	32752.00	885.20	3.11	1.87e-08 ***
	Batch	1	624.00	624.10	2.20	0.14
	Genotype: Batch	35	11954.00	341.60	1.20	0.21
	Residuals	381	108288.00	284.20		

DF: Degrees of freedom, Sum Sq: Sum of squares, Mean Sq: Mean square.

Seedling stage cold tolerance-specific genotyping for *qSCT1* with snpOS00403, snpOS00404, snpOS00406, and snpOS00407 exhibited distinct allelic variation (Table 6). BR11712-4R-227 carried favorable alleles at all four loci and displayed a moderately tolerant response during seedling-stage, indicating a partial but consistent contribution of *qSCT1*. Similarly, Bhutan and Hbj. B. VI were moderately tolerant and possessed favorable alleles at snpOS00403 and snpOS00407, suggesting incomplete genetic fixation for seedling-stage cold tolerance. Notably, BR8907-B-1–2-CS1–4-CS2-P3-4 and BR8909-B-12–2-CS1–4-CS2-P2-3–2 exhibited strong seedling-stage cold tolerance despite lacking favorable *qSCT1* alleles, indicating the involvement of additional QTLs or alternative genetic control. Conversely, susceptible genotype BRRI dhan28 predominantly carried alternative allelic combinations. Most importantly, the susceptible check BR1 possessed favorable alleles for all four SNPs of *qSCT1*, suggested that the *qSCT1* haplotype alone is insufficient to confer cold tolerance under the climatic landscape of Bangladesh, and additional loci or favorable allelic interactions are necessary for functional expression of resilience to cold stress.

### Cold tolerance at the reproductive stage

Under cold stress conditions, all the genotypes flowered between 19 February and 28 March 2024 (S8A Fig in S1 File), indicating that panicle initiation (PI) to booting occurred from the third week of January to the third week of February. During this period, temperatures ranged from 8.7°C to 31.4°C, with an average of 19.8°C (S9A Fig in S1 File). Notably, between 15 and 30 January, daily averages remained below 20°C, with maximum between 18.4°C and 26.9°C, suggesting exposure to natural low temperatures during critical reproductive stages.

Genotypes flowered between 15 and 30 February, including BR12266-44-11-32-5-1-1-HR10-B, BR11303-5R-156, BR11318-5R-106, BR10317-5R-25, Bhutan, BRRI dhan74, BRRI dhan28, BRRI dhan88, IR18A1859, and TP16199, likely encountered severe cold stress at PI and booting stages. Additionally, entries flowering in early March (e.g., Rata Boro, IR83222-F11-173, Hbj. B. VI, Tepi Boro, Mineasahi, and several BR11894 derivatives) experienced moderate cold stress (19.1°C–21.3°C), particularly during booting.

In contrast, all breeding lines in non-stress plots flowered between 17 March and 10 April 2024 (S7B Fig in S1 File). The non-stress plots recorded temperatures between 21.1°C and 25.4°C (max: 26.3°C–31.4°C) from 15 February to 10 March (S9B Fig in S1 File), indicating favorable thermal conditions during reproductive development (PI to booting stage).

Under natural field conditions, all the phenotypic traits except vegetative stage score (VegS) showed a significant response to cold stress (Table 7). Although effective tiller number was significantly affected under both cold stress and non-stress conditions, it was excluded from further analysis. Specifically, in the cold stress plots, young seedlings were damaged by night birds shortly after transplanting, creating vacant spaces in the field. These gaps allowed the remaining plants to compensate by producing more tillers, resulting in a higher effective tiller number under cold stress relative to non-stress conditions. Therefore, traits like PDS, PES, SFS, plant height, days to heading, and panicle length were used for further analysis. Under phytotron conditions, these traits have a significant effect on cold stress, whereas plant height trait didn’t exhibit any significant effect on cold stress (Table 7). Thus, the traits that had a significant effect on cold stress were used for further analysis.

**Table 7:** Effect of cold Stress and nonstress conditions on different agronomic and cold-related traits under natural field and artificial cold screening facilities.

Trait	Mean_cold stress	Mean_non-stress	P-value	t_statistic	Significance
**Natural field conditions**					
Vegs	4.92	4.60	0.14	1.51	ns
PDS	3.19	1.42	0.00	5.63	***
PES	3.89	1.77	0.00	4.96	***
SFS	5.27	3.54	0.00	7.29	***
Plant height (cm)	97.07	104.05	0.00	−3.77	***
Days to heading (days)	163.70	149.71	0.00	11.85	***
Effective tiller number	20.39	14.81	0.00	5.03	***
Panicle length (cm)	21.73	23.93	0.00	−5.97	***
**Artificial cold screening facilities**					
PDS	1.15	1.03	0.03	2.26	*
PES	2.35	1.67	0.00	4.38	***
SFS	5.11	4.16	0.02	2.43	*
Plant height (cm)	97.15	98.44	0.39	−0.87	ns
Days to heading (days)	113.52	108.09	0.00	7.87	***
Panicle length (cm)	21.60	22.81	0.01	−2.95	**

***: P < 0.001, **: P < 0.01, *: P < 0.05, ns: Not significant (P-value>= 0.05).

VegS: Vegetative score, PDS: Panicle degeneration score, PES: Panicle exsertion score, SFS: Spikelet fertility score.

The variation in phenotypic performance of the diverse genotypes based on measured agronomic traits from non-stress to stress conditions under natural (field) and artificial cold screening facilities (phytotron) was represented by the reduction rate in Table 8. The lowest plant height reduction (PHR) was observed in TP16199 (G13) (−10.88% in field). BRRI dhan74 (G21) and IR100723-B-B-B-B-61 (G15) exhibited the least delay in heading date (DHD) (3.50 and 0.57 days) in field and phytotron conditions, respectively. Minimum panicle length reduction (PLR) was observed in BRRI dhan92 (G26) (−4.26% in field) and BR11712-4R-227 (G8) (−16.71% in phytotron). Whereas, the highest PHR was shown in BR10317-5R-25 (G7) (21.85% in the field). The longest DHD was observed in Mineasahi (G5) (25.50 days in the field) and BR11318-5R-106 (G10) (10.07 days in the phytotron). IR100722-B-B-B-B-11 (G14) and BR11894-R-R-R-R-329 (G17) showed the highest PLR (21.96 and 15.49%) under field and phytotron conditions, respectively.

**Table 8:** Reduction rate of the measured characters of diverse panel genotypes due to cold stress under field and phytotron conditions.

Geno type	Designation	Field			Phytotron	
		PHR	DHD	PLR	DHD	PLRR
G1	Rata Boro	−2.00	12.00	−3.17	10.72	12.42
G2	Tepi Boro	0.35	18.00	9.27	4.91	9.12
G3	Mineasahi	−9.96	25.50	−2.22	9.73	11.31
G4	Bhutan	−0.32	5.50	4.41	1.05	7.93
G5	IR83222-F11-173 (Tol. ck.)	3.89	13.00	7.07	0.73	−6.62
G6	Hbj. B. VI	6.36	13.50	−1.22	5.89	−3.95
G7	IR100722-B-B-B-B-11	16.37	20.50	21.96	7.80	10.72
G8	IR100723-B-B-B-B-61	0.77	9.50	0.07	0.57	0.09
G9	BR10317-5R-25	21.85	10.00	15.70	4.69	14.47
G10	BR11303-5R-156	−2.40	20.00	8.87	4.86	−2.01
G11	BR11318-5R-106	6.43	11.50	16.00	10.07	1.24
G12	BR11712-4R-227	4.87	20.50	8.58	2.74	−16.71
G13	BR12266-44-11-32-5-1-1-HR10-B	3.52	4.50	19.73	7.65	16.18
G14	BR8899-14-4-1-2-2-1	15.39	12.00	11.97	5.85	−18.35
G15	TP16199	−10.88	6.00	12.50	1.69	6.87
G16	IR18A1859	7.04	7.50	21.76	4.91	2.52
G17	BRRI dhan28 (Sus. ck.)	19.27	10.50	6.96	3.61	6.87
G18	BRRI dhan67 (Mod. tol. ck.)	11.07	19.50	10.00	2.86	13.19
G19	BR11894-R-R-R-R-169	0.33	15.00	10.15	4.71	15.44
G20	BR11894-R-R-R-R-329	3.81	16.50	15.27	5.80	15.49
G21	BR11894-R-R-R-R-105	6.74	15.00	5.82	8.13	6.31
G22	BR11894-R-R-R-R-110	18.98	13.50	4.82	1.32	7.01
G23	BRRI dhan74	9.70	3.50	5.58	2.07	−1.05
G24	BRRI dhan88	11.70	13.25	17.38	4.19	4.41
G25	BRRI dhan81	13.73	16.50	9.71	3.98	6.54
G26	BRRI dhan92	7.60	11.00	−4.26		

PHR (Plant height reduction), DHD (Delay in heading date), and PLR (Panicle length reduction).

**Fig 3:**
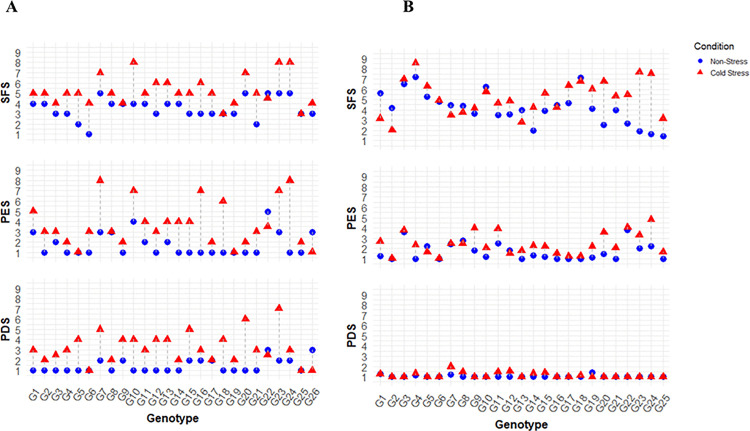
Paired dot plot showing performance of diverse rice germplasm under cold stress and nonstress conditions based on cold-related traits in A) field and B) phytotron conditions. PDS: Panicle degeneration score, PES: Panicle exsertion score, and SFS: Spikelet fertility score. Where, 1 = Highly tolerant, 3 = Tolerant, 5 = Moderately tolerant, 7 = Moderately susceptible, 9 = Susceptible.

The variation of genotypes in cold-related traits like PDS, PES, and SFS under stress and non-stress conditions in the field and phytotron was represented as paired dot plots, comparing where non-stress (blue circle) and cold stress (red triangle) scores for a given entry (Fig 3**).** A longer line indicates a greater difference between the two conditions, suggesting higher susceptibility to cold stress. Conversely, a shorter line indicates a smaller difference, implying better tolerance to cold stress. In the field, the lowest cold trait scores (indicating tolerance) were observed in Bhutan, BRRI dhan67, and BRRI dhan92 (1, PDS); BR11894-R-R-R-R-110, Mineasahi, and BRRI dhan92 (1, PES); and IR83222-F11-173 (1, SFS) (Fig 3A).

In the phytotron, most entries exhibited low degeneration; IR83222-F11-173 and Bhutan performed the lowest PES (1), and Bhutan also showed the lowest SFS score (1; Fig 3B). While BRRI dhan92 demonstrated cold tolerance in the field, this might be attributed to its longer growth duration, avoiding stress conditions. Conversely, Rata Boro and IR83222-F11-173 showed reverse results in the phytotron. SFS was higher under non-stress conditions than cold stress in the phytotron due to high-temperature stress at the reproductive stage in non-stress pots. The ambient temperature for non-stress pots at the flowering stage was 27.6-38.1℃, i.e., higher than 35℃ (S10B Fig in S1 File). So, nonstress plots suffered from heat stress at the reproductive stage, leading to spikelet sterility.

Spearman correlation analysis was performed to elucidate the relationships among the different phenotypic variables at two different levels of cold stress (field and phytotron conditions), and the results are presented in Table 9. Under field, SFS exhibited a significant positive correlation with PDS and PES, indicating that spikelet fertility was associated with panicle degeneration and exsertion. A significant positive correlation was also found between plant height and panicle length. Days to heading was negatively correlated with SFS, suggesting that longer growth duration was associated with lower spikelet fertility. In the case of phytotron conditions, the traits of plant height, days to heading, and panicle length were significantly and positively correlated with each other. Days to heading showed a significant negative correlation with SFS, consistent with field observations.

**Table 9:** Spearman correlation matrix among the phenotypic variables at different levels of cold stress.

	Field_cold stress					
	PDS	PES	SFS	Plant height	Days to heading	Panicle length
**PDS**	1	0.34	0.57**	-0.18	-0.41*	-0.32
**PES**	0.08	1	0.51**	-0.14	-0.28	0.08
**SFS**	-0.16	0.18	1	-0.24	-0.21	-0.26
**Plant height**	0.15	-0.3	0.04	1	0.36	0.57**
**Days to heading**	0.11	-0.38	-0.41*	0.45*	1	0.36
**Panicle length**	0.39	-0.08	-0.22	0.40*	0.44*	1
**Phytotron_cold stress**						
**: Significant at P < 0.01, *: Significant at P < 0.05						

PDS: Panicle degeneration score, PES: Panicle exsertion score, SFS: Spikelet fertility score.

**Fig 4:**
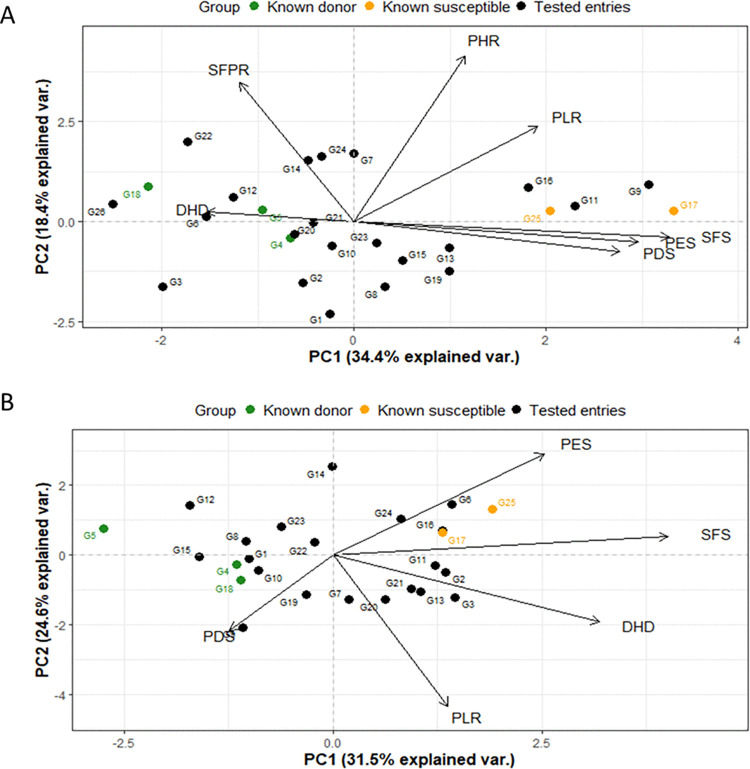
PCA analysis (PC1, PC2) showing traits and genotypes distribution regarding cold tolerance at reproductive stages in rice under A) field and B) phytotron conditions. G1: Rata Boro, G2: Tepi Boro, G3: Mineasahi, G4: Bhutan, G5: IR83222-F11-173 (Tol.ck), G6: Hbj. B. VI, G7: IR100722-B-B-B-B-11, G8: IR100723-B-B-B-B-61, G9: BR10317-5R-25, G10: BR11303-5R-156, G11: BR11318-5R-106, G12: BR11712-4R-227, G13: BR12266-44-11-32-5-1-1-HR10-B, G14: BR8899-14-4-1-2-2-1, G15: TP16199, G16: IR18A1859, G17: BRRI dhan28 (Sus. Ck.), G18: BRRI dhan67 (Mod. tol.Ck.), G19: BR11894-R-R-R-R-169, G20: BR11894-R-R-R-R-329, G21: BR11894-R-R-R-R-105, G22:BR11894-R-R-R-R-110, G23: BRRI dhan74, G24: BRRI dhan88, G25: BRRI dhan81, G26: BRRI dhan92.

Principal Component Analysis (PCA) was conducted to explore the phenotypic variation and reduce the dimension among 25 rice genotypes under cold stress at the reproductive stage under the two environments (field and phytotron conditions). Under field conditions, the first two principal components (PC1 and PC2) explained 57.8% of the total variance, where PC1 accounted for 39.1% of the variability (Fig 4A). Whereas, under controlled phytotron conditions, PC1 and PC2 explained 31.5% and 24.6% of the total variance, summing to 56.1% (Fig 4B). Across both environments, G17 (BRRI dhan28), a known susceptible cultivar, consistently clustered with other susceptible genotypes on the positive side of PC1 for relevant cold traits (such as PDS, PES, SFS, PHR, and PLR in the field and PES, SFS, PLR, and DHD in the phytotron).

Genotyping results for the reproductive stage cold screening revealed clear differentiation between the most cold-tolerant and most cold-susceptible genotypes based on the allelic composition of *qPSST3, qPSST7, and qPSST9* (Table 10). The genotypes Rata Boro, Bhutan, and BR11712-4R-227 possessed partial alleles of *qPSST3, qPSST7, qPSST9*, and *qPSST6*, whereas IR83222-F11-173 carried partial alleles of *qPSST3, qPSST7, and qPSST9*. Although these genotypes exhibited tolerance to cold stress at the reproductive stage, genotypic analysis indicated that none of them possessed the complete set of alleles associated with *qPSST3, qPSST7, and qPSST9*. This differences between phenotypic performance and allelic composition suggests that additional QTLs, alleles, or specific SNP positions beyond *qPSST3, qPSST7, and qPSST9* contributed to reproductive-stage cold tolerance.

**Table 10:** Allelic variation of qPSST3, qPSST7, and qPSST9 among cold-tolerant and cold-susceptible rice genotypes under reproductive-stage cold stress.

ID	Designation	Repro. screening results	qPSST3_1	qPSST3_2	qPSST3_3	qPSST7_1	qPSST7_2	qPSST7_3	qPSST7_4	qPSST9_1	qPSST9_2	qPSST9_3	qPSST9_4	qPSST9_5	qPSST6_1	qPSST6_2	Waxy
			snpOS00815	snpOS00816	snpOS00817	snpOS00818	snpOS00819	snpOS00820	snpOS00821	snpOS00822	snpOS00823	snpOS00824	snpOS00825	snpOS00826	snpOS01157	snpOS01158	snpOS00445
G1	Rata Boro	Tolerant	G:G	A:A	G:G	C:C	A:A	T:T	C:C	G:G	C:C	C:C	G:G	C:C	A:A	G:G	T:T
G4	Bhutan	Tolerant	G:G	G:G	G:G	C:C	A:A	T:T	C:C	G:G	C:C	C:C	G:G	C:C	A:A	G:G	T:T
G5	IR83222-F11-173	Tolerant	G:G	G:G	G:G	C:C	A:A	T:T	C:C	G:G	T:T	C:C	G:G	C:C	C:C	A:A	T:T
G12	BR11712-4R-227	Tolerant	G:G	A:A	T:T	C:C	A:A	T:T	C:C	A:A	T:T	T:T	A:A	C:C	A:A	G:G	C:C
G23	BRRI dhan74	Tolerant	A:A	G:G	G:G	T:T	G:G	C:C	A:A	A:A	C:C	T:T	A:A	T:T	C:C	A:A	C:C
G22	BR11894-R-R-R-R-110	MT	A:A	G:G	G:G	T:T	G:G	C:C	A:A	G:G	C:C	?	G:G	T:T	C:C	A:A	C:C
G8	IR100723-B-B-B-B-61	MT	A:A	G:G	G:G	T:T	G:G	C:C	A:A	G:G	T:T	T:T	A:A	T:T	?	?	T:T
G13	BR12266-44-11-32-5-1-1-HR10-B	MT	A:A	A:A	T:T	?	G:G	C:C	A:A	A:A	C:C	?	A:A	C:C	C:C	A:A	C:C
G15	TP16199	MT	A:A	G:G	G:G	T:T	G:G	C:C	A:A	G:G	C:C	C:C	A:A	T:T	C:C	A:A	C:C
G19	BR11894-R-R-R-R-169	MT	A:A	G:G	G:G	T:T	G:G	C:C	A:A	A:A	T:T	C:C	G:G	T:T	C:C	A:A	C:C
G21	BR11894-R-R-R-R-105	MT	A:A	A:A	T:T	T:T	G:G	C:C	A:A	?	?	?	?	?	?	?	C:C
G10	BR11303-5R-156	MT	A:A	A:A	T:T	T:T	G:G	C:C	A:A	A:A	C:C	C:C	A:A	T:T	C:C	A:A	C:C
G18	BRRI dhan67	MT	A:A	A:A	T:T	T:T	G:G	C:C	A:A	A:A	C:C	C:C	A:A	T:T	C:C	A:A	C:C
G9	BR10317-5R-25	Susceptible	G:G	A:A	T:T	T:T	G:G	C:C	A:A	G:G	T:T	C:C	G:G	T:T	C:C	A:A	C:C
G11	BR11318-5R-106	Susceptible	A:A	A:A	T:T	T:T	G:G	C:C	A:A	A:A	C:C	T:T	A:A	C:C	C:C	A:A	C:C
G17	BRRI dhan28	Susceptible	A:A	A:A	G:G	T:T	G:G	C:C	A:A	A:A	C:C	T:T	A:A	T:T	C:C	A:A	C:C
G25	BRRI dhan81	Susceptible	A:A	A:A	T:T	C:C	A:A	T:T	C:C	A:A	C:C	T:T	A:A	T:T	C:C	A:A	C:C
G24	BRRI dhan88	Susceptible	A:A	A:A	T:T	T:T	G:G	C:C	?	A:A	C:C	?	A:A	T:T	C:C	A:A	C:C

MT: Moderately tolerant. Alleles highlighted in green and light pink represent favorable and unfavorable alleles for specific SNP, respectively.

Under both field and phytotron conditions, PC1 is the primary descriptor of sensitivity, with sensitivity associated with positive values of SFS, PES and possibly PDS. PC2 was explained mostly by height-related parameters PHR and PLR, which may or may not be directly linked to cold stress. These traits could represent growth characteristics that are either indirectly affected by cold or simply vary independently.

However, a notable difference was observed that PDS and DHD were associated with PC1 under field conditions, but contributed more strongly to PC2 under phytotron conditions. This suggests that PDS and DHD may reflect different physiological responses depending on environmental condition. Importantly, another key discrepancy between field and phytotron conditions was DHD contribute to PC1 in the field, while PC2 under phytotron conditions. This divergence highlights the influence of environmental condition on trait expression and underscores the need to interpret PCA results within the framework of specific growing conditions.

To support and validate the PCA-based grouping of genotypes, hierarchical clustering was performed using Euclidean distance and Ward’s method under both field and phytotron conditions. The resulting clusters showed strong concordance with PCA, particularly in the consistent placement of cold-tolerant genotypes in Cluster I and the susceptible check BRRI dhan28 (G23) in Cluster IV across both environments (S13A and S13B Figs in S1 File).

Cluster mean values (S2 Table in S1 File) were used to rank genotypes based on trait performance under field and phytotron conditions (S3 Table in S1 File). The summed scores ranged from 18 to 38, with genotypes with lower summed scores across traits were classified as tolerant (18–22), while those with higher scores were considered as susceptible (33–38). The tolerant genotypes identified were Bhutan, IR83222-F11-173, BR11712-4R-227, BRRI dhan74, and Rata Boro. Genotypes which were grouped into moderately tolerant included, BR11894-R-R-R-R-110, BRRI dhan67 (Mod. tol. ck.), BR11894-R-R-R-R-105, BR11303-5R-156, TP16199, IR100723-B-B-B-B-61, BR12266-44-11-32-5-1-1-HR10-B, and BR11894-R-R-R-R-169. Susceptible genotypes were BRRI dhan88, BRRI dhan28 (Sus. ck.), IR18A1859, BR11318-5R-106, BRRI dhan81, and BR10317-5R-25.

### Performance of genotypes evaluated at both seedling and reproductive stage

**Fig 5:**
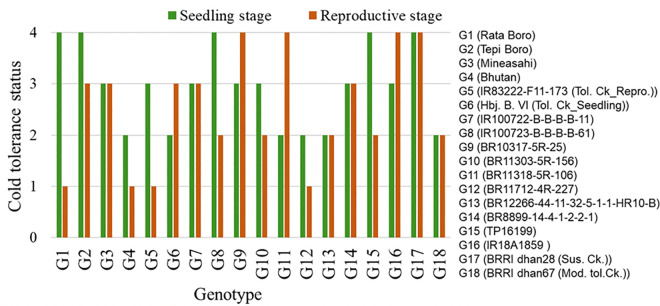
Performance of common 18 diverse rice panel under cold stress at seedling and reproductive stages. Bar heights represent the cold tolerance status for each genotype. Status was defined as: 1 = Tolerant, 2 = Moderately tolerant, 3 = Moderately susceptible, and 4 = Susceptible.

The performance of 18 common rice genotypes evaluated under both seedling and reproductive stages revealed distinct patterns of cold stress tolerance. The full spectrum of observed responses for each genotype at both developmental stages is presented in Fig 5. Genotypes such as Bhutan, BR11712-4R-227, BR12266-44-11-32-5-1-1-HR10-B, and BRRI dhan67 consistently exhibited moderately tolerant to tolerant phenotypes at both stages, indicating stable performance under cold stress.

A few genotypes, including Rata Boro, BR100723-B-B-B-B-61, BR11303-5R-156, TP16199, and IR83222-F11-173, showed tolerance specifically at the reproductive stage but were moderately susceptible to susceptible at the seedling stage. In contrast, BR11318-5R-106 was moderately tolerant only at the seedling stage but susceptible at the reproductive stage. Genotypes like Mineasahi, BR100722-B-B-B-B-11, BR8899-14-4-1-2-2-1, BR10317-5R-25, IR18A1859, Tepi Boro, and the susceptible check BRRI dhan28 showed poor performance across both stages.

Spearman correlation analysis between seedling and reproductive stage results (Table 11) revealed a weakly positive but non-significant correlation between cold tolerance scores at the seedling and reproductive stages (r^2^ = 0.18, P = 0.479), indicating no statistically meaningful association between tolerance levels across these two developmental phases. Thus, seedling and reproductive stage cold tolerance should be considered as separate traits and quite likely under distinct genetic control.

**Table 11:** Spearman correlation for cold tolerance at seedling and reproductive stages in a diverse rice panel.

	Seedling Stage	Reproductive Stage
**Seedling Stage**	1	0.18 ^NS^
**Reproductive Stage**	0.18 ^NS^	1

Cold tolerance was coded as: 1= Tolerant, 2= Moderately tolerant, 3= Moderately susceptible, 4 = Susceptible.

**Fig 6:**
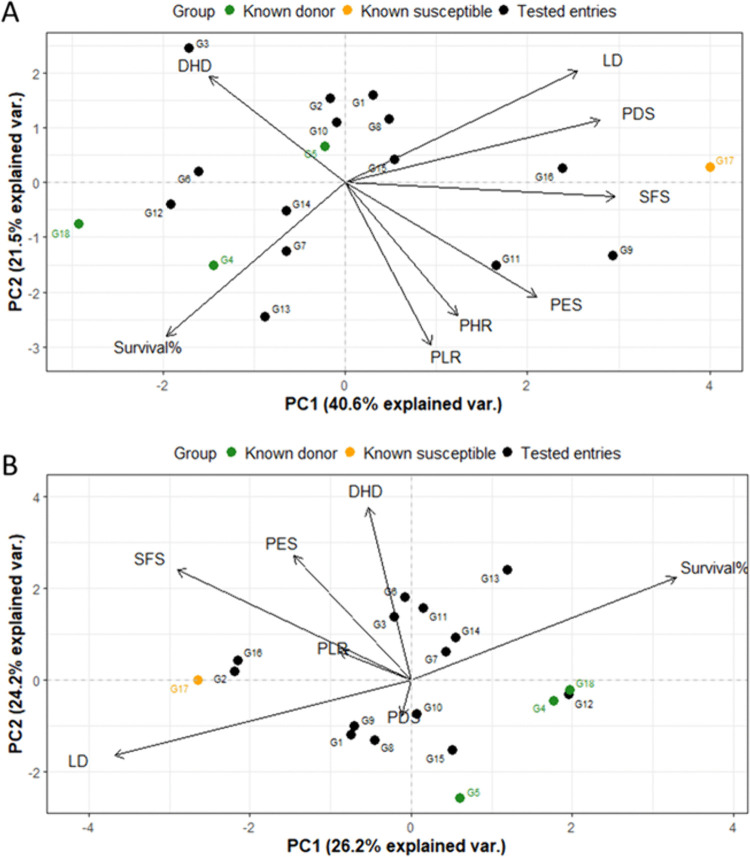
PCA biplot of 18 rice genotypes based on cold stress-related traits at seedling and reproductive stages under A) field and B) phytotron conditions. G1: Rata Boro, G2: Tepi Boro, G3: Mineasahi, G4: Bhutan, G5: IR83222-F11-173 (Tol.ck), G6: Hbj. B. VI, G7: IR100722-B-B-B-B-11, G8: IR100723-B-B-B-B-61, G9: BR10317-5R-25, G10: BR11303-5R-156, G11: BR11318-5R-106, G12: BR11712-4R-227, G13: BR12266-44-11-32-5-1-1-HR10-B, G14: BR8899-14-4-1-2-2-1, G15: TP16199, G16: IR18A1859, G17: BRRI dhan28 (Sus. Ck.), G18: BRRI dhan67 (Mod. tol.Ck.).

A PCA was employed to investigate the relationship among cold stress-related traits at both the seedling (LD, survival percentage) and reproductive stages (PDS, PES, SFS, PHR, DHD, PLR) across 18 common rice genotypes, under field and phytotron conditions (Fig 6).

Under field conditions (Fig 6A), the first two principal components (PCs) accounted for 62.1% of the total variation, with PC1 contributing 40.6%. PC1 had a greater contribution of LD, PDS, and SFS, higher values of these traits corresponding to cold susceptibility. On the other hand, PLR, PHR, DHD and survival percentage contributed to PC2. Thus, a combination of seedling and reproductive traits contributed to each of the first two principal components.

Under phytotron conditions (Fig 6B), PC1 and PC2 explained 50.4% of the total variation (PC1: 26.2%, PC2: 24.2%). PC1 was mostly dominated by seedling traits LD and survival%, with some contribution from SFS, while PC2 was mostly controlled by reproductive traits DHD, PES and SFS. Thus, phytotron data shows distinct partitioning of PC1 and PC2 between seedling and reproductive-stage traits, supporting the hypothesis that seedling and reproductive-stage tolerance are separate traits having different genetic control.

## Discussion

Cold stress adversely affects rice productivity, with its varying effects depending on growth stage as well as the severity and duration of exposure. Rice plants are susceptible to cold damage at any developmental phase – including germination, seedling, vegetative, reproductive, and maturity stages [11,21]. This study investigates a diverse set of rice genotypes under cold conditions during the seedling and reproductive stages, aiming to elucidate stage-specific vulnerabilities and to reinforce the importance of comprehensive cold screening at all growth stages to unravel the complex resilience pathways to cold stress.

Cold damage at the seedling stage manifests primarily as leaf yellowing due to inhibited chlorophyll synthesis, stunted growth, and eventual seedling mortality. These physiological disruptions often reduce photosynthetic efficiency and elevate respiration, leading to assimilate shortages that impair tissue development and halt growth. LD is widely used to differentiate cold-tolerant genotypes from susceptible ones; however, it alone does not fully capture the complexity of seedling-stage cold tolerance [19].

Therefore, survival rate is also considered a critical metric for identifying promising donor genotypes [21]. In this study, 38 diverse rice genotypes were evaluated for seedling stage cold tolerance across three batches (B1, B2, B3) using both LD and survival percentage. Two genotypes, BR8907-B-1–2-CS1–4-CS2-P3-4 (G36) and BR8909-B-12–2-CS1–4-CS2-P2-3–2 (G37) consistently demonstrated superior tolerance across all batches, with the lowest LD scores (3.7 and 3.9) and highest survival rates (34.4−37%). These lines, derived from three-way crosses involving donor parents 89010-TR1232-4-1 and CUNJING15 under BRRI dhan29 and BRRI dhan28 backgrounds, respectively, represent valuable resources for breeding programs. The performance of the tolerant and susceptible varieties aligned with their expected tolerance categories (Fig 3), justifying the tolerance levels of G36 and G37. Also, six genotypes- BR11318-5R-106 (G11), BR11712-4R-227 (G12), BR12266-44-11-32-5-1-1-HR10-B (G13), Bhutan (G4), BRRI dhan58 (G43), and BRRI dhan89 (G45) were classified as moderately tolerant, showing LD scores of 4.9-6.6 and survival rates of 14.1−31%. Among these, Bhutan and BR12266-44-11-32-5-1-1-HR10-B showed relatively high survival (24.3-24.7) despite moderate LD, indicating possible recovery or stress avoidance mechanisms. This partial independence of LD and survival percentage suggests distinct physiological pathways governing cold tolerance for these two traits in some genotypes. Suh et al. (2013) reported LD scores ranging from 3 to 8 (mean value of 5) among 23 elite cultivars grown under cold-water irrigation, reinforce LD as a visual assessment of damage, while survival percentage offers direct measure of resilience. The strong negative correlation observed between LD score and survival percentage (Spearman’s r = −0.708, p < 0.0001) indicates these two parameters are closely related and can, at least partially, substitute for each other in assessing seedling-stage cold tolerance. Genotypes with low LD and high survival are reliably tolerant, while outliers may possess unique genetic and physiological mechanisms worth further investigation.

Seedling-stage cold tolerance in rice is a quantitatively inherited trait governed by multiple QTLs, including *qSCT1*, with individual loci contributing partial effects to overall tolerance [5,7,25] (Table 6). The occurrence of cold-tolerant genotypes lacking favorable alleles of *qSCT1* further supports the involvement of additional loci underlying seedling-stage cold tolerance. Genome-wide association studies have also revealed multiple QTLs and favorable haplotypes contributing to seedling cold tolerance and functional QTL fine-mapping has led to the discovery of specific cold tolerance genes such as *OsbZIP72* [26] and cold stress response loci including *COLD11* and *OsCTS11* [27]. These findings reinforce the polygenic architecture of seedling-stage cold tolerance and support the idea that, in addition to major QTLs like *qSCT1*, other genetic components contributed to phenotypic variation observed among tolerant genotypes.

Reproductive-stage cold stress in rice was evaluated under field and phytotron conditions to assess genotype performance across environments. Screening at young microspore stage using cold air, water and natural winter conditions enabled consistent evaluation [28]. The trait values were normalized as reduction rates (e.g., PHR, PLR, DHD) relative to controls [21,29], along with visual injury scores (PDS, PES, and SFS), to quantify sterility and panicle development failure (Table 8; Fig 3A and 3B). These remain standard indicators for reproductive stage cold tolerance [21,30].

Significant phenotypic variation among the genotypes across environments revealed diverse cold response mechanisms. Genotypes like Bhutan, IR83222-F11-173, Rata Boro, and BRRI dhan74 showed consistently low reduction rates and injury scores, suggesting physiological resilience to cold stress. By contrast, the genotypes TP16199, BR11303-5R-156, and BR12266-44-11-32-5-1-1-HR10-B showed moderate cold tolerance scoring and moderate level of reduction in the studied traits, while several genotypes (BR11894-R-R-R-R-110, BR11303-5R-156, Rata Boro, IR83222-F11-173, and BRRI dhan67) exhibited delayed heading (12-20.50 days) but maintained low PHR, PLR, PDS, PES, and SFS, suggesting escape of cold stress in these genotypes due to the delayed heading. However, the opposite result was reported by Suh et al. (2010) [7,31]. Thus, DHD alone may not be a reliable measure for screening reproductive-stage cold-tolerant rice [32].

Correlation analysis revealed that growth reduction traits (PHR, PLR, DHD) were positively associated, as were traits related to injury scores (PDS, PES, SFS) (Table 9), while negative correlations were observed between injury score and growth reduction traits, indicating that greater reduction in growth traits aligns with higher injury. This observation is consistent with the findings [21,31] that cold stress reduces culm length and panicle development, ultimately impairing spikelet fertility. PCA distinguished the genotype responses, with DHD contributing to PC1 in field condition implying delayed flowering as a cold escape strategy. Under phytotron condition, DHD explained by PC2. This distinct shift in DHD’s contribution between the two environments highlights a significant Genotype×Environment interaction, suggesting that the phenotypic traits governing cold stress variability differ based on growth conditions (Fig 4).

Comparable studies [29] have validated the Average Tolerance Index (ATI) for rice germplasm assessment under cold stress at early growth stages emphasizing genotype × environment interactions in trait expression.

Hierarchical clustering and trait-based ranking demonstrated stable performance of genotypes such as IR83222-F11-173, Bhutan, Rata Boro, and BRRI dhan74 across both environments, suggesting robust cold tolerance (S12 Fig, S3 Table in S1 File). This validates previous claims that cultivars tolerant under controlled cold conditions also perform well under field stress [30], supporting phytotron screening as a reliable proxy. Eighteen genotypes evaluated at both seedling and reproductive stages showed a weak and non-significant correlation between the two stages indicating different genetic mechanisms governing cold tolerance at each stage. This is supported by studies showing distinct QTLs and physiological responses control tolerance at seedling (e.g., *qCTS12.1*) and reproductive stages (e.g., *qPSR10*) [5,12]. For example, early-stage traits such as germination percentage, shoot and root length, and coleoptile length are strongly intercorrelated, but non-significant and negligible positive association between the germination and early seedling establishment [29]. Together these results emphasize the need to screen germplasm for cold tolerance at both stages, rather than assuming tolerance at one stage will predict tolerance for another.

The known QTLs *qPSST3, qPSST7,* and *qPSST9* were reported to confer reproductive-stage cold tolerance [7]. However, genotyping results (Table 10) indicated that these loci alone did not fully account for the observed tolerance in the evaluated 12 genotypes. Many tolerant lines possessed only partial alleles, reflecting a weak association between these markers and phenotypic performance. This supports the view that reproductive-stage cold tolerance is a polygenic trait, influenced by multiple minor-effect QTLs and stage-specific regulatory mechanisms [33]. Consequently, reliance on a limited set of *qPSST*-linked markers is insufficient for effective marker-assisted selection. Comprehensive approaches, including high resolution QTL mapping, fine mapping, and identification of key SNPs, are necessary to uncover additional genomic regions and improve the precision of breeding for cold-tolerant rice adapted to low-temperature environments.

PCA combining both developmental stages under field conditions indicated seedling and reproductive traits may share similar physiological pathways, supporting the hypothesis that certain genotypes possess integrated tolerance mechanisms operating across the developmental stages. This is consistent with recent studies suggesting that cold stress responses involve shared signaling pathways – such as ABA-mediated regulation, ROS detoxification, and membrane stabilization – active during both vegetative and reproductive phases [34]. However, under phytotron conditions, seedling and reproductive traits contributed to different components, supporting partial independence and stage-specific genetic control. These observations align with previous reports showing that different QTLs contribute to cold tolerance at seedling and reproductive stages, highlighting the complex nature of cold tolerance at different stages [21]. Supporting this finding, a comprehensive study was carried out by Kim et al. (2025), integrated QTL mapping with transcriptome analysis at the budding and seedling stages, revealed distinct differences in cold tolerance mechanisms between developmental stages [35]. Together, these findings suggest that while certain cold-responsive pathways may be shared, independent regulatory mechanisms act at specific growth stages, emphasizing the need for stage-specific screening approaches for accurate classification of genotypes and effective breeding for cold tolerance.

## Conclusions

In this study, BR8907-B-1–2-CS1–4-CS2-P3-4 and BR8909-B-12–2-CS1–4-CS2-P2-3–2 exhibited consistent cold tolerance at seedling stage, Rata Boro, BR100723-B-B-B-B-61, BR11303-5R-156, TP16199, and IR83222-F11-173 demonstrated tolerance to cold stress at reproductive-stage and Bhutan, BR11712-4R-227, and BR12266-44-11-32-5-1-1-HR10-B exhibited moderate to strong tolerance across both stages, indicating presence of stage-specific differential cold-stress response among these genotypes. Genotyping results and phenotypes under cold stress also indicated genetic control in these genotypes is independent and tolerance expressed at one stage does not necessarily confer tolerance at another, underscoring the importance of stage-specific cold screening. Integrating stage-specific tolerant donors into a single genetic background is recommended to develop rice varieties resilient to cold stress across multiple developmental phases, thereby stabilizing yields under low-temperature conditions.

## Supporting information

S1 File **S1 Fig.** Growth stage-specific low temperature response associated with physiological and morphological impairment during seedling to flowering stage in rice. **S2 Fig**. Seeding of pregerminated seeds in seedling trays for cold screening at the seedling stage. **S3 Fig.** Seedlings at three-leaf stage prepared for placing in the cold water tank for artificial cold screening at the seedling stage. **S4 Fig.** Seedlings in the cold water tank adjusted at 13℃ for artificial cold screening at the seedling stage. **S5 Fig**. Temperature profile of batch 1, 2 and 3 in cold water tank. **S6 Fig.** (A) Onset of cold stress symptoms in seedlings; (B) Seedlings at the scoring stage (8th day), when the susceptible check died. **S7 Fig.** (A) Leaf discoloration (LD) scoring based on SES score, (B) Cold water treated trays after 7 days of cold stress. **S8 Fig.** Dot plot showing flowering window of the diverse panel under A) early sown cold stress plot (CS) and B) regular Boro season (NS) plot in field. **S9 Fig**. Maximum and minimum temperature of field conditions under A) cold stress, B) Non stress conditions. **S10 Fig.** Temperature profile of air and water in artificial cold screening facilities (CSF) of A) cold stress, and B) ambient air temperature. **S11 Fig.** Cold treated plants at PI to booting stage in phytotron for reproductive stage cold screening. **S12 Fig**. Tiller which was at meiotic stage was labeled and treated under cold stress condition. **S13 Fig.** Dendrogram showing the hierarchical clustering of rice genotypes based on phenotypic traits under (A) field and (B) phytotron conditions with cold stress at the reproductive stage. **S1 Table.** Phenotypic performance in term of LD score and survival rate (%) of 38 rice genotypes after cold stress (13°C) at seedling stage. **S2 Table.** Cluster mean of different cold traits in the clusters formed at different cold levels using Euclidian distance matrix. **S3 Table**. Ranking of diverse rice germplasm for their relative cold related traits at reproductive stage in a cluster analysis using Euclidian distance matrix.
(RAR)
